# Three decades of progress and setbacks since the first international conference on population and development

**DOI:** 10.2471/BLT.24.291654

**Published:** 2024-04-01

**Authors:** Manjulaa Narasimhan, Lale Say, Pascale Allotey

**Affiliations:** a Department of Sexual and Reproductive Health and Research, World Health Organization, 20 Avenue Appia, 1211 Geneva, Switzerland.

Thirty years ago, the International Conference on Population and Development in Cairo, Egypt, affirmed the right of all couples and individuals to attain the highest standard of sexual and reproductive health;^1^ 10 years later, the World Health Organization (WHO) published its reproductive health strategy.^2^

Both^3^ underscored the centrality of comprehensive sexual and reproductive health and rights for realizing the right to health and enabling all individuals to contribute equally to societies and economies. Grounded in gender equality, these frameworks are supportive of women as rights holders and recognize the immutability of sexual health.

Comprehensive sexual and reproductive health and rights across the life course encompass the full spectrum of related services, from safeguarding girls from harmful traditional practices to providing comprehensive sexuality education that equips young people with essential knowledge for navigating relationships free from stigma, discrimination, violence and coercion. These rights also include supporting menstrual health; contraception and fertility care; prevention, management and treatment of sexually transmitted and reproductive tract infections; prevention of unsafe abortion; respectful care during pregnancy and childbirth; and sexual health and well-being beyond reproductive years.

All these efforts have resulted in substantial progress. The proportion of women aged 15–49 years who use a modern contraceptive method doubled from an estimated 467 million in 1990 to 874 million women in 2022,^4^ and contraceptive options have expanded. As of March 2022, 60% of WHO Member States (117/194) have included the human papillomavirus vaccine in their routine national immunization schedules.^5^ Evidence-based medical abortion regimens are expanding global access to abortion,^6^ and more than 60 countries have liberalized abortion laws in the past 30 years.^7^

However, notable improvements are beset by uneven^8^ and stagnating progress. Despite reductions in maternal deaths globally from 2000 to 2015,^9^ maternal mortality stalled or even increased from 2016 to 2020 – with particularly high rates in a few countries, and growing inequalities within countries.^9^ These trends reflect gender inequality, economic disparities, structural discrimination, displacement and conflict, as well as insufficient progress towards universal health coverage (UHC).^10^

Realizing sexual and reproductive health and rights is an imperative; here we suggest several next steps for this unfinished agenda. 

First, opportunities exist to further strengthen human rights-based approaches and frameworks in sexual and reproductive health, generate evidence of their effectiveness and embed human rights principles including non-discrimination. Work to maintain these rights in humanitarian crises, conflict settings, responses to natural disasters and infectious disease outbreaks is needed.

Second, better-functioning health systems that take a primary health-care approach have the potential to promote equity and deliver expanded access to comprehensive, quality sexual and reproductive health services, thereby enhancing UHC.^11^ Effective integration of sexual and reproductive health in primary health care includes ensuring that: (i) related services are part of essential health service and health benefit packages; (ii) relevant supplies and equipment are in place; (iii) competent health workers are available, supported by regulatory mechanisms for prescribing, and with a defined scope of practice for various sexual and reproductive health services; and (iv) evidence-based clinical guidelines are implemented consistently.^11^

Third, new technologies and alternative approaches to service delivery, including multipurpose products and self-care interventions such as self-tests and self-monitoring devices, have the potential to enhance sexual and reproductive health and rights by expanding access to and choice of quality health care, as well as autonomy of end-users.^12^

Fourth, while robust science underlies global progress on sexual and reproductive health and rights, many evidence-based interventions depend on modifying behaviours, shifting harmful norms and driving social change. Expanded implementation research is therefore needed to translate evidence into impact. Research clarifying the economic case for fulfilling these rights could also be beneficial.

The health sector has a pivotal role to play in realizing sexual and reproductive health and rights. Yet many determinants of sexual and reproductive health emanate from outside the health sector. We need renewed, concerted and multisectoral action on comprehensive sexual and reproductive health and rights for all.
